# Mycobacterium haemophilum Infection in a Patient With Systemic Lupus Erythematosus and Antisynthetase Syndrome: A Case Report and Literature Review

**DOI:** 10.7759/cureus.99909

**Published:** 2025-12-23

**Authors:** Ana Luís Vasconcelos, Luís Neves da Silva, Carlos Capela, Rosário Araújo

**Affiliations:** 1 Internal Medicine, Hospital de Braga, Braga, PRT; 2 Internal Medicine, Life and Health Sciences Research Institute, School of Medicine, University of Minho, Braga, PRT

**Keywords:** inflammatory idiopathic myopathies, lymphopenia, mycobacterium haemophilum, skin ulcer, systemic lupus erythematosus

## Abstract

Nontuberculous mycobacteria infections exhibit a broad and heterogeneous clinical spectrum, predominantly affecting immunocompromised individuals, particularly those with CD4^+^ T-lymphocyte depletion. We report the case of a 61-year-old woman with systemic lupus erythematosus and antisynthetase syndrome, receiving treatment with mycophenolate mofetil (MMF) and prednisolone, who presented with small erythematous, papular, and exudative skin lesions involving both legs and feet. Direct staining revealed atypical acid-fast bacilli, and polymerase chain reaction identified *Mycobacterium haemophilum*. Within a few weeks, new lesions developed, progressing to extensive ulceration. Empirical triple therapy with azithromycin, moxifloxacin, and rifabutin was initiated, while MMF was discontinued and prednisolone tapered to 5 mg daily. Intravenous immunoglobulin (IVIG) was introduced to control immune disease activity. Gradual clinical improvement was achieved over the following months. This case highlights the challenges of managing *M. haemophilum* infection while balancing infection control and immunomodulation, and supports IVIG as a safe adjunctive therapeutic option.

## Introduction

*Mycobacterium haemophilum* is a rare, slow-growing, nontuberculous mycobacteria (NTM) that prefer a lower growth temperature, around 28-30°C, and an iron-supplemented culture, making it unique among other Mycobacterium species [1]. Since its first description in 1978, approximately 300 cases have been reported in the literature, although the true epidemiology is likely underestimated due to limited speciation and diagnostic challenges [2,3]. It primarily causes localized or disseminated infections in immunocompromised hosts and is only rarely identified in immunocompetent individuals.

In recent years, the incidence of NTM infections has been rising, particularly among older adults and patients with underlying structural lung disease. Advances in diagnostic methods have also made these organisms easier to identify, and our understanding of their true global epidemiology continues to evolve, especially for rarer species such as *M. haemophilum*. CD4+ T lymphocytes play a central role in host defense against NTM, and their depletion, whether due to human immunodeficiency virus (HIV), idiopathic CD4+ lymphopenia, or immunosuppressive therapies, significantly increases susceptibility. Cytokine interactions among macrophages, T lymphocytes, and natural killer cells are critical for granuloma formation. Accordingly, immunosuppressive therapies, particularly tumor necrosis factor-alpha inhibitors, are associated with an increased risk of NTM infections. More rarely, genetic defects in interferon γ or interleukin 12 may predispose to disease [3].

This case report describes a rare *M. haemophilum* infection in a patient with complex immune-mediated diseases. Our objective is to highlight the diagnostic and clinical challenges posed by this specific infection in immunosuppressed hosts and to provide insights that may support clinicians facing similar scenarios.

## Case presentation

**Figure 1:**
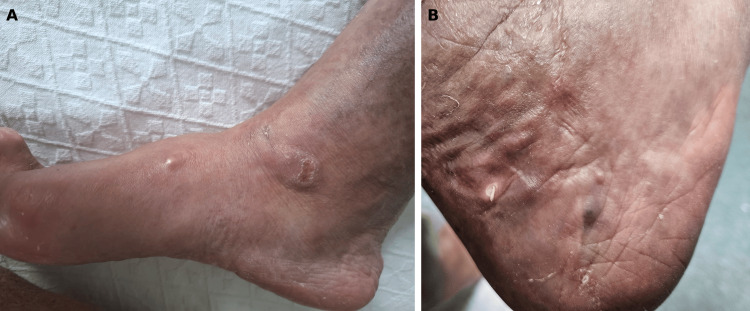
Initial cutaneous lesions on the right foot (A) Two small erythematous nodules on the medial side of the foot. (B) A small pustule on the back of the ankle

A 61-year-old woman presented to our tertiary hospital with erythematous, papular and exudative skin lesions involving both legs and feet. She had been followed at our institution for over 25 years since her diagnosis of systemic lupus erythematosus (SLE). After an initial episode of lupus nephritis with a complete response to treatment, her disease activity remained low while she was on daily hydroxychloroquine 400 mg and prednisolone 5 mg. Three years before, she was diagnosed with antisynthetase syndrome (ASS), marked by Jo-1 and Ro-52 antibody positivity, myopathy, “mechanic’s hands,” arthritis, and a nonspecific interstitial pneumonia. At that time, treatment was started with mycophenolate mofetil (MMF), titrated up to 2 g daily. Over the past year, worsening myalgias led to an increase in prednisolone to 20 mg daily. One month before her current presentation, she was treated with flucloxacillin for presumed cellulitis of her left leg. The initial lesions were erythematous papules, about 1 cm in diameter, which progressively enlarged, ulcerated, and discharged exudative material (Figure 1).

Laboratory evaluation revealed profound lymphopenia (270/µL) with a CD4+ T-cell count of 58/µL and an inverted CD4/CD8 ratio. Hemoglobin and platelet counts were normal. Inflammatory markers were low (erythrocyte sedimentation rate 14 mm/hour, ferritin 205 ng/mL, and C-reactive protein 14 mg/dL). Autoimmune activity markers were unremarkable, with a negative double-stranded DNA antibody and normal complement levels (C1q, C3, and C4). Serum protein electrophoresis and immunofixation showed no monoclonal spike, and immunoglobulin levels (IgG, IgM, and IgA) were normal. Renal function was preserved, and a 24-hour urine collection showed no significant proteinuria. Creatine kinase, myoglobin, and aldolase were mildly elevated but below her baseline values. Infectious serologies for common pathogens, including HIV, hepatitis A, B, and C viruses, syphilis, *Borrelia burgdorferi*, varicella-zoster virus, cytomegalovirus, and measles, did not indicate recent infection. Blood cultures, including fungal-specific cultures, were negative. All the laboratory test results are shown in Table 1.

**Table 1:** Laboratory test results CLIA: novel chemiluminescent immunoassay for Treponema pallidum; CMV: cytomegalovirus; HAV: hepatitis A virus; HIV: human immunodeficiency virus; HCV: hepatitis C virus; VZV: varicella-zoster virus; HBs: hepatitis B surface antibody; HBc: hepatitis B core

Parameters	Patient values	Reference range
Hemoglobin	11.0 g/dL	11.9-15.6 g/dL
Mean corpuscular volume	87.9 fL	82.9-98.0 fL
Mean corpuscular hemoglobin concentration	31.6 g/dL	31.8-34.7 g/dL
Leukocytes	5.1 × 10^3^/uL	4.0-11.0 × 10^3^/uL
Neutrophils	4.7 × 10^3^/uL	1.8-7.1 × 10^3^/uL
Lymphocytes	0.27 × 10^3^/uL	1.2-3.4 × 10^3^/uL
Platelets	181 × 10^3^/uL	150-500 × 10^3^/uL
Lymphocyte flow cytometry		
CD4 total	58/uL	-
CD8 total	167/uL	-
CD4/CD8	0.35	-
Erythrocyte sedimentation rate	14 mm/hour	1-20 mm/hour
Ferritin	205 ng/mL	10-291 ng/mL
C-reactive protein	14 mg/dL	<5.0 mg/dL
Antinuclear antibodies	Negative (1/80)	Negative ≤1/80
Anti-double-stranded DNA antibodies	Negative (23.5 UI/mL)	Negative <30 UI/mL
C1q	15 mg/dL	10-22 mg/dL
C3	124 mg/dL	90-180 mg/dL
C4	28 mg/dL	10-40 mg/dL
Serum protein electrophoresis and immunofixation	Absence of monoclonal spikes	-
IgG	674 mg/dL	650-1,600 mg/dL
IgM	184 mg/dL	50-300 mg/dL
IgA	107 mg/dL	40-350 mg/dL
Urea	60 mg/dL	19-49 mg/dL
Creatinine	1.1 mg/dL	0.60-1.10 mg/dL
Urine sediment	0-2 leukocytes, erythrocytes and epithelial cells	-
24-hour urine proteinuria	0.4 g	<0.2 g
24-hour urine albuminuria	5 mg	<30 mg
Creatine kinase	726 U/L	34-145 U/L
Myoglobin	945 ng/mL	<110 ng/mL
Aldolase	15 U/L	1-7.5 U/L
Anti-HIV (fourth-generation test)	Negative	-
Anti-HAV total antibody	Positive (>100)	Negative <20 mIU/mL
Anti-HAV IgM antibody	Negative (0.20)	Negative <0.80 index
Anti-Hbs antigen	Negative (0.28)	Negative <1.0 index
Anti-Hbs antibody	Positive (193.76 mIU/mL)	Positive >10 mIU/mL
Anti-Hbc antibody	Negative (0.8)	Negative <1.0 index
Anti-HCV antibody	Negative (0.1)	Negative <0.2 index
CLIA (syphilis)	Nonreactive	-
Anti-Borrelia IgG antibody	Negative	Negative <5000 UA/mL
Anti-Borrelia IgM antibody	Negative	Negative <2000 UA/mL
Anti-VZV IgG antibody	Positive (1,611 mUI/mL)	Positive >150 mUI/mL
Anti-VZV IgM antibody	Negative (0.308)	Negative <1.0 index
Anti-CMV IgG antibody	Positive (>30 U/mL)	Positive >30 U/mL
Anti-CMV IgM antibody	Negative (0.22 U/mL)	Negative <0.6 U/mL
Anti-measles IgG antibody	Positive (>300 UA/mL)	Positive >16.50 UA/mL
Anti-measles IgM antibody	Negative (0.20 index)	Negative <0.90 index
Blood cultures (three sets anaerobic and aerobic, one set for fungal culture)	Negative	-

**Figure 2:**
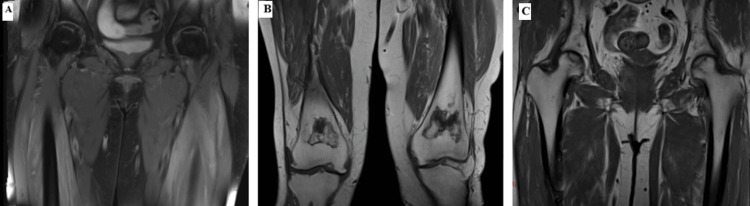
Magnetic resonance imaging of the lower limbs (A) T2-weighted MRI coronal image of the proximal hip muscles showing high signal within the muscles with slight muscle enlargement. (B) T1-weighted MRI coronal image of the distal femurs showing bilateral medullary areas surrounded by a serpiginous low-signal rim, with a central fatty signal, compatible with bone infarcts. (C) T1-weighted MRI coronal image of the femoral head showing peripheral areas of medullary bone surrounded by a peripheral low sign, suggestive of bilateral avascular osteonecrosis MRI: magnetic resonance imaging

Transthoracic echocardiography was normal, and magnetic resonance imaging of the lower limbs showed muscle edema, as well as osteonecrosis of the femoral heads and knees, without evidence of deep tissue infection (Figure 2).

**Figure 3:**
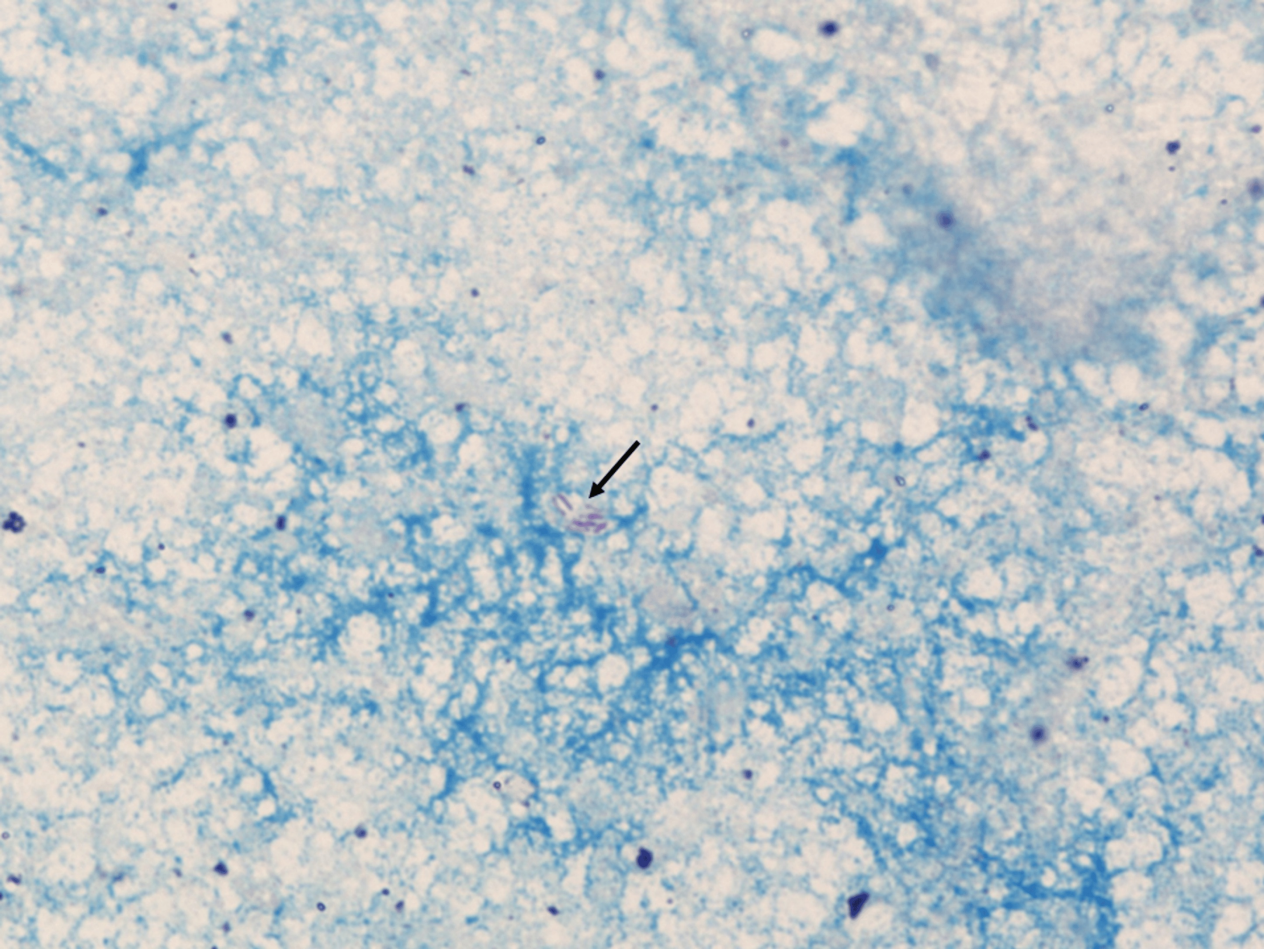
Ziehl-Neelsen staining of the purulent material Ziehl-Neelsen staining (Kinyoun method), at 1,000× magnification, shows numerous atypical red acid-fast bacilli (arrow) against a blue background

Purulent material was obtained through incision of two nodular lesions. Direct Gram stain and microbiological culture were negative. Ziehl-Neelsen staining revealed atypical acid-fast bacilli, morphologically consistent with NTM or Nocardia spp. (Figure 3).

A 16S rRNA polymerase chain reaction (PCR) followed by sequencing identified *M. haemophilum*. Cultures on Lowenstein-Jensen medium remained negative after 42 days. Despite molecular identification of the species, it was not possible to culture the sample in enriched media. Drug susceptibility testing could not be performed, as it requires adequate growth, and molecular resistance assays for *M. haemophilum* are unavailable in our country.

A triple empirical regimen with azithromycin 500 mg, moxifloxacin 400 mg, and rifabutin 300 mg daily was started without significant adverse effects. To prevent *Pneumocystis jirovecii *infection, atovaquone was begun, chosen over cotrimoxazole due to its lower risk of bone marrow suppression. MMF and prednisolone doses were gradually reduced. To control SLE and ASS activity without further immunosuppression, intravenous immunoglobulin (IVIG) was initiated at 2 g/kg every four weeks.

**Figure 4:**
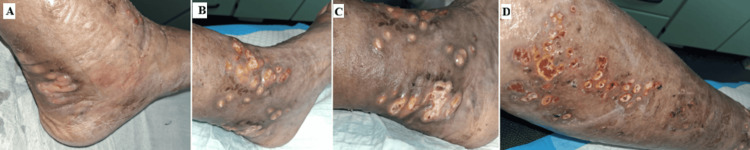
Cutaneous lesions during the second and third months (A) Multiple, coalescent papules on the back of the right ankle that later released purulent material and ulcerated. (B,C) Progression of multiple, coalescent papules that extended from the foot to the leg and ulcerated after releasing exsudative material. (D) Multiple ulcers extending from the foot to the leg in a sporotrichoid distribution

During the first month of treatment, the number of lesions remained stable. However, in the second and third months, there was a rapid and exuberant progression, with multiple new, larger nodules and pustules (Figure 4).

**Figure 5:**
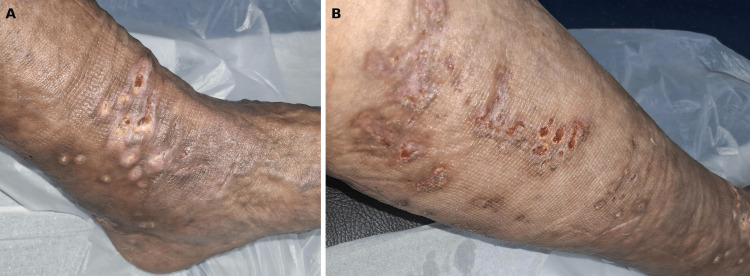
Cutaneous lesions on the left leg during the sixth month (A) Small, nonexudative ulcers in regression. (B) Small ulcers that are almost completely scarred Smaller ulcers with evident signs of scarring

At this point, MMF was discontinued, and prednisolone was maintained at 5 mg daily, based on the assumption of uncontrolled infection caused by severe lymphopenia. We also considered the possibility of a paradoxical reaction, an immune reconstitution phenomenon often reported in the related infection caused by *Mycobacterium ulcerans*. In the following months, lesions regressed, showing significant clinical improvement (Figure 5).

Despite stopping MMF and tapering prednisolone, along with immune disease control using IVIG, lymphopenia continued, although the CD4+ T cell count increased to 153/µL. After six months of treatment, most ulcerated lesions were healing with scarring, although some inflammatory lesions persisted. A prolonged treatment period of 12-24 months is expected, considering the difficulties of NTM treatment and ongoing immunodeficiency. The IVIG dose was reduced to the lowest effective dose to manage immune disease activity.

## Discussion

*M. haemophilum* is an environmental NTM found ubiquitously in soil and water. Despite widespread exposure, clinically significant infection remains rare, usually occurring in individuals with impaired cell-mediated immunity. Reported manifestations include skin and soft-tissue infection, pulmonary disease, lymphadenitis, and bone and joint infections. With advances in HIV therapy, the affected adult population has shifted, and most cases now occur in patients receiving immunosuppressive treatment for immune-mediated diseases or following solid-organ or hematopoietic stem-cell transplantation [4].

Cutaneous involvement is the most common presentation, with a wide clinical spectrum that may include erythematous papules, plaques, nodules, necrotic abscesses, chronic ulcers, and purpuric or annular lesions. These lesions resemble those caused by *M. ulcerans* and *M. marinum*, species with which *M. haemophilum* shares genomic similarities [1]. An acral distribution is typical, reflecting its predilection for cooler temperatures [5].

Laboratory diagnosis is challenging due to the organism’s unique growth requirements. Standard mycobacterial culture techniques are inadequate, and visible growth may take up to eight weeks. The addition of ferric ammonium citrate or hemin to solid egg-based media, such as Lowenstein-Jensen, or to solid agar-based media, such as Middlebrook, is essential for proper growth. Molecular detection of mycobacterial DNA by PCR is a rapid diagnostic test that is becoming widely used [1,4].

Treatment should ideally be guided by antimicrobial susceptibility testing, although this is not feasible for all NTM species and all antimycobacterial agents. In vitro and in vivo susceptibilities may differ, as susceptibility testing protocols are not standardized worldwide [1,6]. Dual or triple empirical regimens are generally recommended, and most experts advise combining a quinolone, a macrolide, and a rifamycin. *M. haemophilum* is usually resistant to ethambutol and isoniazid [1]. Other agents, including amikacin, doxycycline, linezolid, or trimethoprim-sulfamethoxazole, may be considered in specific clinical scenarios [5]. We selected a triple regimen of azithromycin, moxifloxacin, and rifabutin, representing the three main antimycobacterial classes active against this species and providing a simple daily regimen of one pill each. The duration of antimicrobial therapy remains undefined and is influenced by disease presentation, the degree of immunosuppression, and clinical response. Reported treatment durations range from 6 to 24 months, with cure being the most common outcome [1].

Given the rarity of *M. haemophilum* infections in patients with SLE and idiopathic inflammatory myopathies, including ASS, dermatomyositis, and polymyositis, we reviewed case reports published in the literature (Table 2).

**Table 2:** Cases of Mycobacterium haemophilum infection in patients with systemic lupus erythematosus and idiopathic inflammatory myositis DM: dermatomyositis; SLE: systemic lupus erythematosus; ABA: abatacept; AZA: azathioprine; BEL: belimumab; CCT: corticosteroid; CP: cyclophosphamide; CsA: cyclosporine A; LEF: leflunomide; HCQ: hydroxychloroquine; MTX: methotrexate; MMF: mofetil mycophenolate; TAC: tacrolimus; TAL: thalidomide; AK: amikacin; AZI: azithromycin; CI: ciprofloxacin; CLR: clarithromycin; DC: D-cycloserine; DOX: doxycycline; E: ethambutol; IMI: imipenem; I: isoniazid; LE: levofloxacin; MXF: moxifloxacin; RB: rifabutin; R: rifampicin ^*^Patient on chronic suppression antimycobacterial therapy

Study	Sex/age	Disease	Immunosuppression	Affected organ	Treatment	Outcome	Treatment duration
Teh et al. [7]	F/25	SLE	MMF, CCT	Soft-tissue	CLR, E, I, R	Cure	6 months
Demenj et al. [8]	F/38	SLE	BEL, MMF, TAC, CCT	Skin, soft-tissue	LE, RB, E	Partial cure	13 months
Gao et al. [9]	F/30	SLE	CCT	Skin	CLR, R, MFX	Partial cure	Unknown
Tyner and Wilson [10]	F/52	SLE	CP, AZA, CCT	Skin, joint	AZI, MFX, DOX	Cure	18 months
Rojas-Rojas et al. [11]	F/29	SLE	MMF, CCT	Skin	R, I, E, LE	Cure	Unknown
Tan et al. [12]	F/59	SLE	Unknown	Skin	CLR, CI, R, I, E	Partial cure	Unknown^*^
Su et al. [13]	F/45	SLE	LEF, HCQ, CCT	Skin	R, CLR, MFX	Cure	Unknown
Nookeu et al. [14]	F/25	SLE	Unknown	Skin, bone	IMI, AK; then AZI, CI, R	Relapse	6 months
	F/39	SLE	Unknown	Soft-tissue	AK; then CLR, LE	Cure	6 months
	F/57	SLE	Unknown	Skin	CLR, CI, R	Cure	6 months
	F/39	SLE	Unknown	Skin	AK; then CLR, LE, DC	Cure	12 months
	F/47	SLE, DM	Unknown	Skin	IMI, AK; then AZI, CI, R	Cure	6 months
Yasen et al. [2]	F/61	DM	CP, TAL, HCQ, CsA, CCT	Skin	CLR, R, MFX	Died	-
Harder et al. [15]	F/38	DM	MMF, HCQ, MTX, CCT	Skin	CLR, CI, R	Cure	Unknown
Nishikawa et al. [16]	M/68	PM	MTX, TAC, ABA, CCT	Skin, soft-tissue	RB, AZI, E	Cure (surgery)	12 months
Shih et al. [17]	M/60	PM	CCT	Skin, soft-tissue	E, R, CLR, CI, AK	Died	-
Ferreira et al. [18]	M/67	PM	CCT	Skin	R, CLR, CI	Died	-

In our patient, the appearance of new skin lesions approximately 8-12 weeks after starting antimycobacterial therapy raised concerns about immune reconstitution inflammatory syndrome (IRIS), also known as a paradoxical reaction, as has been well described in *M. ulcerans* disease [19]. This phenomenon occurs due to immune system recovery, where newly competent T cells trigger an exaggerated inflammatory response, especially in the presence of an active infection. Cases of *M. haemophilum* infections complicated by IRIS have mainly been reported in HIV-infected patients following the initiation of antiretroviral therapy [2,10,20,21]. However, a recent review identified four patients with IRIS associated with immune-mediated diseases, namely, SLE, dermatomyositis, polymyalgia rheumatica, and rheumatoid arthritis, who were recovering from pharmacologically induced immunosuppression while being treated for *M. haemophilum* infection [2]. Discontinuation of ongoing immunosuppressive therapy, which led to an increase in CD4+ T-lymphocyte counts, likely triggered this reaction.

In our patient, long-term antimycobacterial therapy is expected, given the low likelihood of full recovery of leukocyte and lymphocyte counts in a patient with persistent cytopenias since being diagnosed with SLE over 25 years ago. Leukopenia, whether due to lymphopenia, neutropenia, or both, is common in SLE, affecting approximately 20% of patients and correlating with disease activity. Mild leukopenia is usually asymptomatic and does not require specific treatment [22]. However, cytopenias are also frequently a consequence of immunosuppressive therapies. MMF exerts a cytostatic effect on B- and T-lymphocytes and may induce secondary hypogammaglobulinemia [23]. Corticosteroids are well known to cause multiple immune defects, most notably CD4+ T-cell lymphopenia. Consequently, opportunistic infections associated with low CD4+ T-cell counts, including NTM infections, are more frequently reported in patients receiving long-term corticosteroid therapy [24].

Our patient had chronic mild leukopenia, probably indicating low SLE activity, which worsened and progressed to severe lymphopenia after starting MMF and increasing corticosteroids. Because CD4+ T cells are crucial for immune defense against NTM, this patient was especially vulnerable.

The urgency of stopping ongoing immunosuppressive therapy to restore lymphocyte counts raised concerns about controlling SLE and ASS activity. One of the main benefits of IVIG is that, unlike traditional immunosuppressants, which increase the risk of infection, it provides passive immunity and may lower susceptibility to opportunistic infections [25]. IVIG is an on-label treatment approved by the European Medicines Agency for severe forms of dermatomyositis, including interstitial lung disease, extensive skin involvement, and dysphagia. In SLE, it has been used off-label, mainly in refractory cases where conventional immunosuppressants are not tolerated or are contraindicated, with reported benefits particularly in hematological, renal, and neuropsychiatric manifestations [26]. Accordingly, considering the dual need to manage immune-mediated disease and treat a potentially severe NTM infection, IVIG was deemed the safest and most effective option. This approach has rarely been documented in the literature [15,27]. Most published case reports of *M. haemophilum* infection in immunosuppressed patients describe simple discontinuation of immunosuppressive therapy [2,8,28-30]. This method appeared unwise in our case because of the coexistence of two immune-mediated diseases, SLE and ASS, with the latter in high activity during the year prior to the infection. An immunomodulatory dose of 2 g/kg, administered over three days, was started according to recommendations for other immune-mediated diseases. A longer course of therapy was expected, given the extended treatment duration needed for NTM infections. This regimen is similar to IVIG use in neurological diseases such as chronic inflammatory demyelinating polyradiculopathy or in dermatomyositis, where long courses of therapy (12 months or more) have shown safety and effectiveness. Regular assessments of clinical response enable dose adjustments, and doses as low as 0.5 g/kg have shown benefit in several immune-mediated conditions [26].

## Conclusions

This case highlights the challenges of managing *Mycobacterium haemophilum* infection amid immunosuppression and active immune-mediated disease. Our experience emphasizes the importance of balancing infection control with immunomodulation and supports IVIG as a safe therapeutic option. We believe this approach contributed to a positive clinical outcome and may help guide clinicians facing similar situations.
